# Crystal structure and Hirshfeld surface analysis of 3-amino-1-oxo-2,6,8-triphenyl-1,2,7,8-tetra­hydro­iso­quinoline-4-carbo­nitrile

**DOI:** 10.1107/S2056989021000785

**Published:** 2021-01-29

**Authors:** Farid N. Naghiyev, Maria M. Grishina, Victor N. Khrustalev, Ali N. Khalilov, Mehmet Akkurt, Anzurat A. Akobirshoeva, İbrahim G. Mamedov

**Affiliations:** a Department of Chemistry, Baku State University, Z. Khalilov str. 23, Az, 1148 Baku, Azerbaijan; b Peoples’ Friendship University of Russia (RUDN University), Miklukho-Maklay St.6, Moscow, 117198 , Russian Federation; c N. D. Zelinsky Institute of Organic Chemistry RAS, Leninsky Prosp. 47, Moscow, 119991 , Russian Federation; d "Composite Materials" Scientific Research Center, Azerbaijan State Economic University (UNEC), H. Aliyev str. 135, Az 1063, Baku, Azerbaijan; e Department of Physics, Faculty of Sciences, Erciyes University, 38039 Kayseri, Turkey; f Acad. Sci. Republ. Tadzhikistan, Kh. Yu. Yusufbekov Pamir Biol. Inst., 1 Kholdorova St, Khorog 736002, Gbao, Tajikistan

**Keywords:** crystal structure, cyclo­condensation product, 1,2,7,8-tetra­hydro­iso­quinoline ring system, Hirshfeld surface analysis

## Abstract

In the title compound, C_28_H_21_N_3_O, the 1,2-di­hydro­pyridine ring of the 1,2,7,8-tetra­hydro­iso­quinoline ring system is planar as expected, while the cyclo­hexa-1,3-diene ring has a twist-boat conformation, with Cremer–Pople parameters *Q*_T_ = 0.367 (2) A, θ = 117.3 (3)° and φ = 327.3 (4)°. The dihedral angles between the best planes through the iso­quinoline ring system and the three phenyl rings are 81.69 (12), 82.45 (11) and 47.36 (10)°. In the crystal, mol­ecules are linked *via* N—H⋯O and C—H⋯N hydrogen bonds, forming a three-dimensional network. Furthermore, the crystal packing is dominated by C—H⋯π bonds with a strong inter­action involving the phenyl H atoms. The role of the inter­molecular inter­actions in the crystal packing was clarified using Hirshfeld surface analysis, and two-dimensional fingerprint plots indicate that the most important contributions to the crystal packing are from H⋯H (46.0%), C⋯H/H⋯C (35.1%) and N⋯H/H⋯N (10.5%) contacts.

## Abstract 2

The supra­molecular structure of the compound is stabilized by a three-dimensional array of N—H⋯O and C—H⋯N hydrogen bonds and C—H⋯π inter­actions.

## Chemical context   

For many decades, considerable inter­est in organic and medicinal chemistry has been directed toward the synthesis of various biologically valuable nitro­gen heterocycles (Mamedov *et al.*, 2019 ▸; Naghiyev, 2019 ▸; Kerru *et al.*, 2020 ▸). They are prevalent structural motifs in many compounds, also having applications in coordination chemistry and material science (Zubkov *et al.*, 2018 ▸; Mahmoudi *et al.*, 2019 ▸; Velásquez *et al.*, 2019 ▸). The majority of tetra­hydro­iso­quinoline moieties containing anti­tumor anti­biotics, such as saframycins, renieramycins, safracins, ecteinascidins, tetra­zomine, lemonomycin, dnacins and aclindomycins, have already been isolated from natural sources and reproduced applying different effective techniques (Scott & Williams, 2002 ▸).

Owing to the above-mentioned value of tetra­hydro­iso­quinolines, there have been significant developments in this class of compounds. Herein, and in the framework of our ongoing structural studies (Naghiyev *et al.*, 2020*a* ▸,*b* ▸,*c* ▸), we report the crystal structure and Hirshfeld surface analysis of the title compound, 3-amino-1-oxo-2,6,8-triphenyl-1,2,7,8-tetra­hydro­iso­quinoline-4-carbo­nitrile.
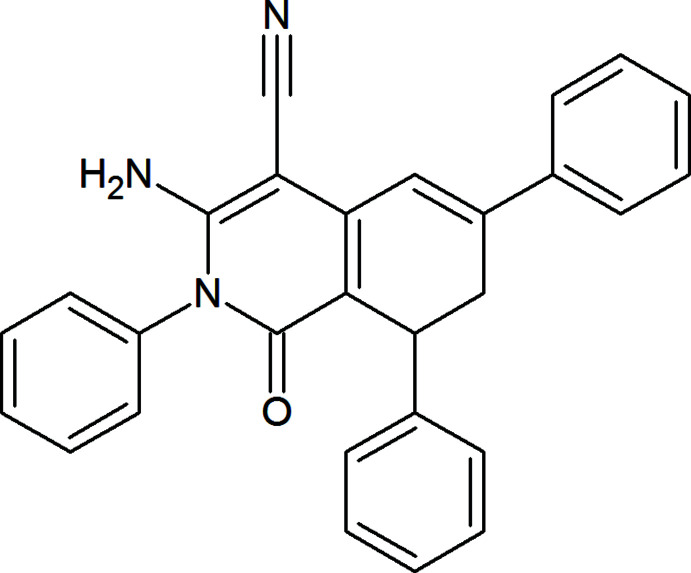


## Structural commentary   

**Figure 1:**
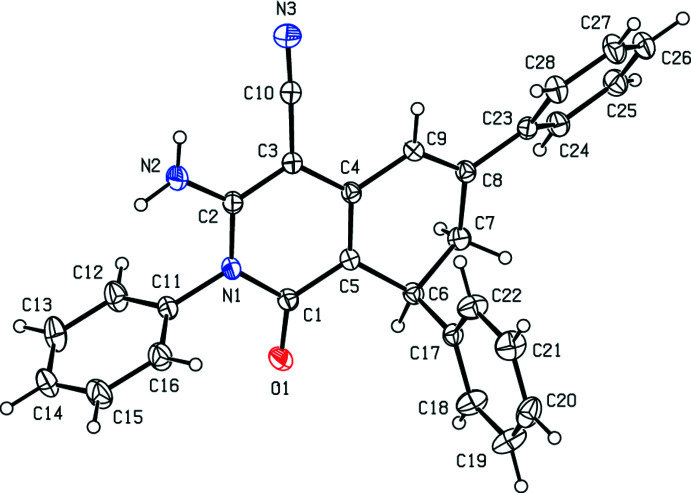
The mol­ecular structure of the title compound. Displacement ellipsoids are drawn at the 30% probability level.

As shown in Fig. 1 ▸, the 1,2-di­hydro­pyridine ring (N1/C1–C5) of the 1,2,7,8-tetra­hydro­iso­quinoline ring system (N1/C1–C9) is planar as expected, while the cyclo­hexa-1,3-diene ring (C4–C9) has a twist-boat conformation, with Cremer–Pople parameters *Q*_T_ = 0.367 (2) Å, θ = 117.3 (3)° and φ = 327.3 (4)°. The dihedral angles between the best planes through the iso­quinoline ring system and the three phenyl rings (C11–C16, C17–C22 and C23–C28) are 81.69 (12), 82.45 (11) and 47.36 (10)°, respectively. All bond lengths (Allen *et al.*, 1998 ▸) and bond angles are all normal.

## Supra­molecular features   

**Figure 2:**
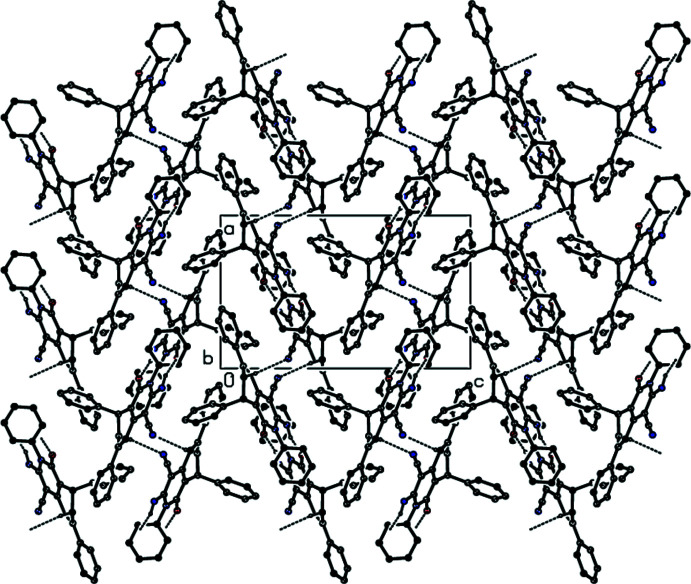
A view of the inter­molecular N—H⋯O and C—H⋯N hydrogen bonds of the title compound down the *b* axis. H atoms not involved in hydrogen bonding have been omitted for clarity.

**Figure 3:**
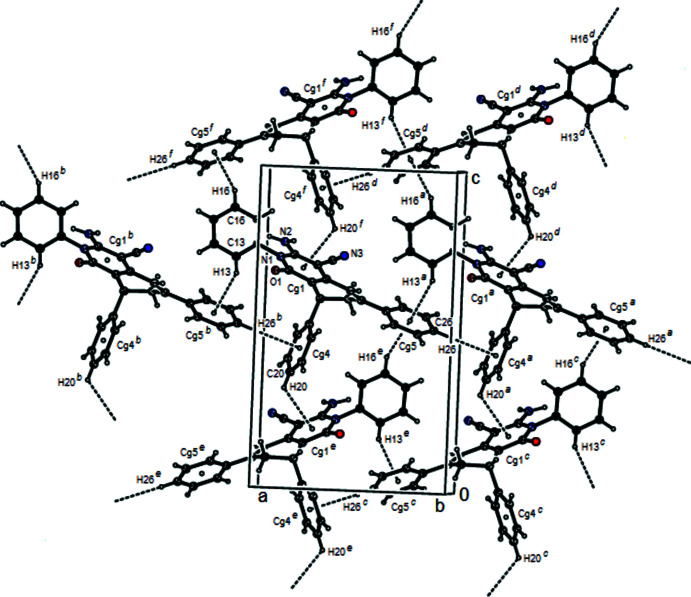
View of the C—H⋯π inter­actions of the title compound down the *b* axis. H atoms not involved in hydrogen bonding have been omitted for clarity. [Symmetry codes: (*a*) −1 + *x*, *y*, *z*; (*b*) 1 + *x*, *y*, *z*; (*c*) 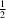 − *x*, 1 − *y*, −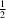 + *z*; (*d*) 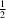 − *x*, 1 − *y*, 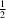 + *z*; (*e*) 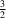 − *x*, 1 − *y*, −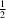 + *z*; (*f*) 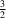 − *x*, 1 − *y*, 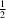 + *z*]. The centroids are defined in Table 1 ▸.

In the crystal, mol­ecules are linked via N—H⋯O and C—H⋯N hydrogen bonds, forming a three-dimensional network (Table 1 ▸, Fig. 2 ▸). Furthermore, the crystal packing is dominated by C—H⋯π inter­actions with a strong involvement of the phenyl hydrogens on C13 (H13) and C26 (H26) (Table 1 ▸, Fig. 3 ▸).

## Hirshfeld surface analysis   

**Figure 4:**
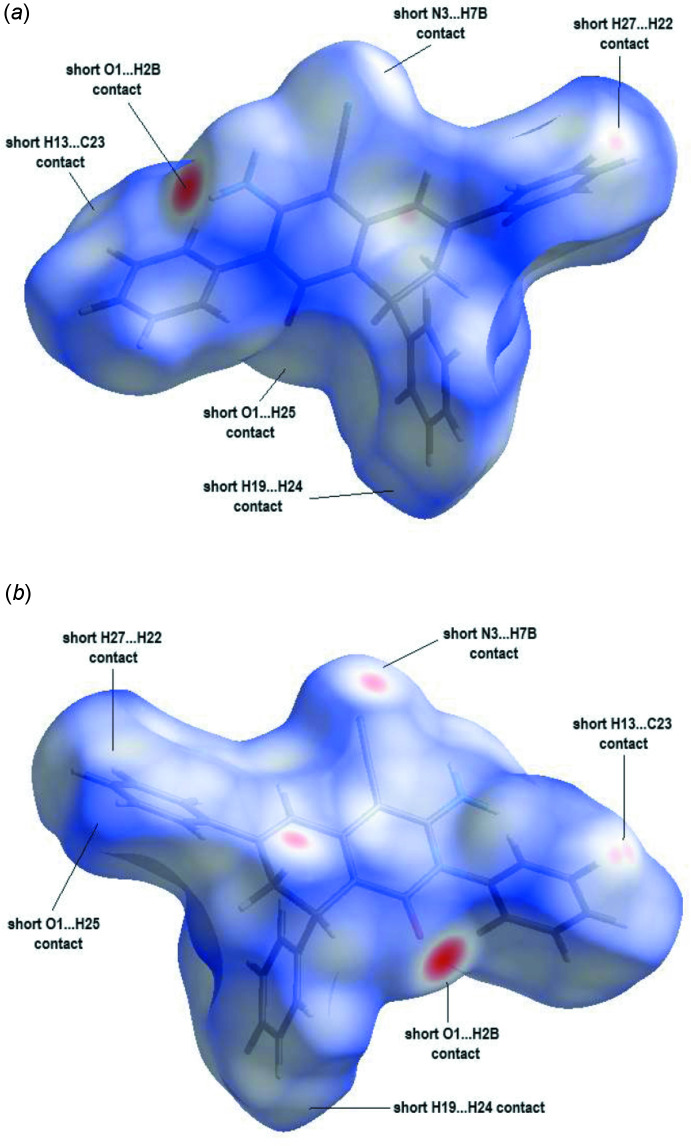
(*a*) Front and (*b*) back sides of the three-dimensional Hirshfeld surface of the title compound plotted over *d*_norm_ in the range −0.4556 to 1.6207 a.u.

**Figure 5:**
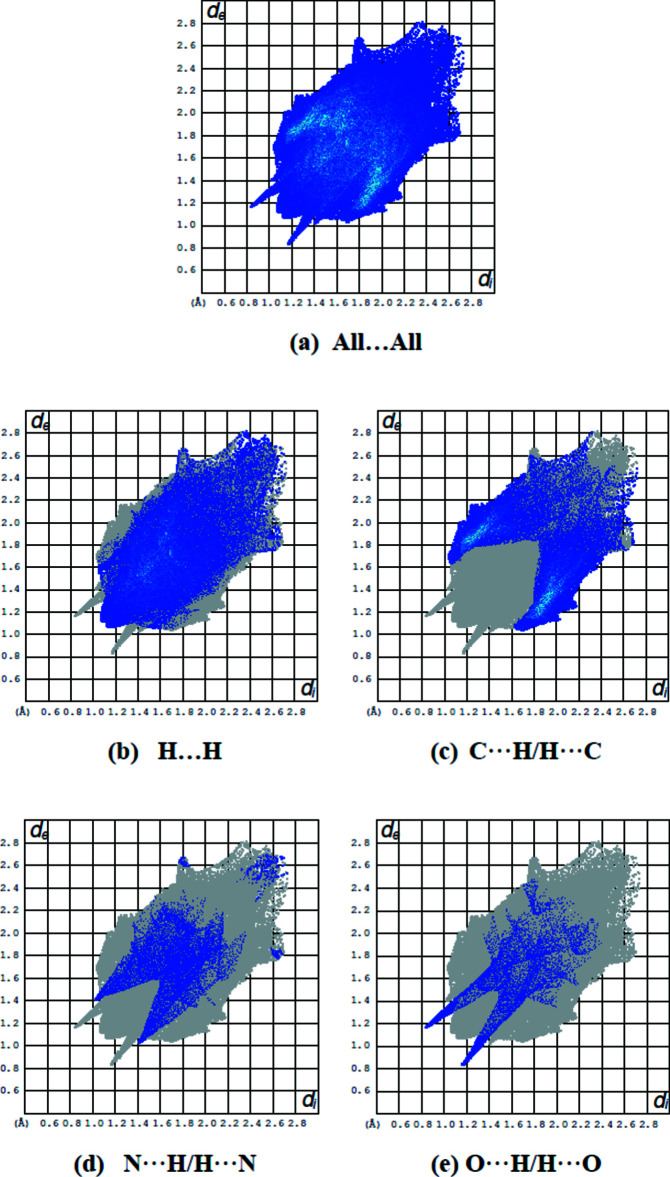
Two-dimensional fingerprint plots of the title compound, showing (*a*) all inter­actions, and delineated into (*b*) H⋯H, (*c*) N⋯H/H⋯N, (*d*) C⋯H/H⋯C and (*e*) O⋯H/H⋯O inter­actions [*d*_e_ and *d*_i_ represent the distances from a point on the Hirshfeld surface to the nearest atoms outside (external) and inside (inter­nal) the surface, respectively].

The Hirshfeld surfaces and two-dimensional fingerprint plots were calculated using *CrystalExplorer* (McKinnon *et al.*, 2007 ▸). Hirshfeld surfaces enable the visualization of inter­molecular inter­actions with different colours and colour intensity representing short or long contacts and indicating the relative strength of the inter­actions. Fig. 4 ▸(*a*) and Fig. 4 ▸(*b*) show the front and back sides of the three-dimensional Hirshfeld surface of the title compound plotted over *d*_norm_ in the range −0.4556 to 1.6207 a.u. Here, the bright-red spots appearing near O1 and N3 result from the N2—H2*B*⋯O1 and C7—H7*B*⋯N3 inter­actions, which play a significant role in the mol­ecular packing of the title compound. The overall two-dimensional fingerprint plot for the title compound and those delineated into H⋯H, C⋯H/H⋯C, N⋯H/H⋯N and O⋯H/H⋯O contacts are illustrated in Fig. 5 ▸, together with their relative contributions to the Hirshfeld surface while details of the various contacts are given in Table 2 ▸. The percentage contributions from the different inter­atomic contacts to the Hirshfeld surfaces are as follows: H⋯H (46.0%), C⋯H/H⋯C (35.1%), N⋯H/H⋯N (10.5%) and O⋯H/H⋯O (6.5%) (Table 3 ▸). The other C⋯N/N⋯C, C⋯C and C⋯O/O⋯C contacts contribute less than 1% to the Hirshfeld surface mapping and have negligible directional impact on the mol­ecular packing (Table 3 ▸).

## Database survey   

A survey of the Cambridge Structural Database (CSD version 5.41, update of March 2020; Groom *et al.*, 2016 ▸) reveals five comparable tetra­hydro­iso­quinoline derivatives, 2-methyl-1,2,3,4-tetra­hydro­iso­quinoline trihydrate (KUGLIK; Lang­en­ohl *et al.*, 2020 ▸), (1*S*,2*R*)-2-[(3*R*,4*S*)-3-methyl-4-phenyl-1,2,3,4-tetra­hydro­isoquinolin-2-yl]-1,2-di­phenyl­ethanol (POPYEB; Ben Ali & Retailleau, 2019 ▸), (3S*,4R*)-4-fluoro-3-(4-meth­oxy­phen­yl)-1-oxo-2-phenyl-1,2,3,4-tetra­hydro­iso­quinoline-4-carb­oxy­lic acid (CARCOQ; Lehmann *et al.*, 2017 ▸), (*S*)-benzyl 3-phenyl­carbamoyl-1,2,3,4-tetra­hydro­iso­quinoline-2-carb­oxy­l­ate (LAQKUL; Naicker *et al.*, 2012 ▸) and 2-[(1*R*,3*S*)-6,7-di­meth­oxy-1-phenyl-1,2,3,4-tetra­hydro­isoquinolin-3-yl]-4-phen­yl-1,3-thia­zole (AZUSOE; Pawar *et al.*, 2011 ▸).

The compound KUGLIK co-crystallizes with three water mol­ecules in the asymmetric unit, which leads to the formation of intense hydrogen bonding in the crystal. In the crystal of POPYEB, mol­ecules are packed in a herringbone manner parallel to (103) and (10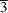) *via* weak C—H⋯O and C—H⋯π(ring) inter­actions. In the crystal of CARCOQ, mol­ecules are linked by an O—H⋯O hydrogen bond, forming chains propagating along the *a*-axis direction. The chains are linked by C—H⋯F hydrogen bonds, forming layers lying parallel to the *ab* plane. In LAQKUL, there are two independent mol­ecules in the asymmetric unit. The heterocyclic ring assumes a twisted boat conformation and N—H⋯O inter­actions help to construct the three-dimensional network within the crystal packing. In AZUSOE, no classical hydrogen bonds nor π–π inter­actions were found in the crystal structure.

## Synthesis and crystallization   

To a solution of 2-acetyl-5-oxo-*N-*3,5-tri­phenyl­penta­namide (5.1 mmol) in aceto­nitrile (40 ml) was added malono­nitrile (5.2 mmol). The solution was stirred for 5 min at room temperature, ethyl­enedi­amine (5.2 mmol) was added and the mixture refluxed for 4 h and cooled down to room temperature. The reaction product precipitated from the reaction mixture as pale-yellow single crystals, was collected by filtration and purified by recrystallization in ethanol/water solution (yield 70%, m.p. 554-556 K).

^1^H NMR (300 MHz, DMSO-*d*_6_): 3.2 (*dd*, ^3^ *J*_H–H_ =9.8 Hz, ^3^ *J*_H–H_ = 2.9 Hz, 2H, CH_2_); 4.25 (*dd*, ^3^ *J*_H–H_ = 9.8 Hz, ^3^ *J*_H–H_ = 2.9 Hz, 1H, CH—Ar); 6.7 (*s*, 2H, NH_2_); 6.8 (*s*, 1H, CH=); 6.9–7.7 (*m*, 15H, arom).

^13^C NMR (75 MHz, DMSO-*d*_6_): 35.17 (CH-Ar), 43.57 (CH_2_), 109.86 (=CH), 117.67 (=C_quat_), 119.52 (CN), 125.96 (2CH_arom_), 126.72 (2CH_arom_), 126.86 (CH_arom_), 127.39 (CH_arom_), 127.99 (2CH_arom_), 128.66 (2CH_arom_), 128.89 (2CH_arom_), 128.89 (2CH_arom_), 129.21 (=C_quat_), 129.56 (CH_arom_), 129.94 (=C_quat_), 135.27 (N—C_ar_), 139.07 (C_ar_), 139.40 (C_ar._), 144.14 (=C_quat_—N), 160.73 (O=C_quat_—N), 167.96 (=C_quat_—Ar).

## Refinement   

Crystal data, data collection and structure refinement details are summarized in Table 4 ▸. The H atoms of the NH_2_ group were located in the difference-Fourier synthesis and refined isotropically with *U*_iso_(H) = 1.2*U*_eq_(N). All C-bound H atoms were positioned geometrically and refined using a riding model, with C—H = 0.93–0.98 Å, and with *U*_iso_(H) = 1.2*U*_eq_(C). Two reflections, (0 1 1) and (1 0 1), affected by the incident beam-stop and owing to poor agreement between observed and calculated intensities, nine outliers, (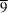 9 7), (9 0 7), (0 6 5), (5 14 12), (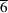 9 2), (1 0 9), (1 13 10), (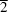 7 15) and (9 9 7), were omitted in the final cycles of refinement. The title compound crystallizes in a non-centrosymmetric space group, *P* 2_1_2_1_2_1_, but the absolute structure could not be determined reliably, and the Flack parameter is inconclusive {Flack *x* = −0.6 (9), determined using 1593 quotients [(*I* ^+^) − (*I* ^−^)]/[(*I* ^+^) + (*I* ^−^)] (Parsons *et al.*, 2013 ▸)}.

## Supplementary Material

Crystal structure: contains datablock(s) I. DOI: 10.1107/S2056989021000785/vm2245sup1.cif

Structure factors: contains datablock(s) I. DOI: 10.1107/S2056989021000785/vm2245Isup2.hkl

Supporting information file. DOI: 10.1107/S2056989021000785/vm2245Isup3.cml

CCDC reference: 2058071

Additional supporting information:  crystallographic information; 3D view; checkCIF report

## Figures and Tables

**Table 1:** Hydrogen-bond geometry (Å, °) *Cg*1, *Cg*4 and *Cg*5 are the centroids of the 1,2-di­hydro­pyridine ring (N1/C1–C5) and the C17–C22 and C23–C28 phenyl rings, respectively.

*D*—H⋯*A*	*D*—H	H⋯*A*	*D*⋯*A*	*D*—H⋯*A*
N2—H2*B*⋯O1^i^	0.90 (3)	2.09 (3)	2.813 (3)	136 (3)
C7—H7*B*⋯N3^ii^	0.97	2.54	3.391 (4)	146
C13—H13⋯*Cg*5^iii^	0.93	2.74	3.576 (3)	149
C26—H26⋯*Cg*4^iv^	0.93	2.83	3.729 (3)	162
C16—H16⋯*Cg*5^v^	0.93	2.97	3.603 (3)	126
C20—H20⋯*Cg*1^vi^	0.93	2.96	3.514 (3)	120

Symmetry codes: (i) 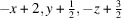; (ii) 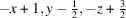; (iii) 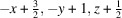; (iv) 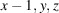; (v) 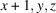; (vi) 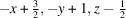.

**Table 2:** Summary of short inter­atomic contacts (Å) in the title compound

Contact	Distance	Symmetry operation
O1⋯H25	2.88	1 + *x*, *y*, *z*
H27⋯H22	2.43	− 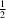 + *x*, 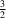 − *y*, 1 − *z*
H13⋯C23	2.86	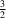 − *x*, 1 − *y*, 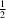 + *z*
H19⋯H24	2.41	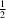 + *x*, 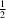 − *y*, 1 − *z*

**Table 3:** Percentage contributions of inter­atomic contacts to the Hirshfeld surface for the title compound

Contact	Percentage contribution
H⋯H	46.0
C⋯H/H⋯C	35.1
N⋯H/H⋯N	10.5
O⋯H/H⋯O	6.5
C⋯N/N⋯C	0.9
C⋯C	0.5
C⋯O/O⋯C	0.4

**Table 4:** Experimental details

Crystal data	
Chemical formula	C_28_H_21_N_3_O
*M*_r_	415.48
Crystal system, space group	Orthorhombic, *P*2_1_2_1_2_1_
Temperature (K)	296
*a*, *b*, *c* (Å)	10.7038 (3), 11.6096 (4), 17.5182 (5)
*V* (Å^3^)	2176.93 (11)
*Z*	4
Radiation type	Mo *K*α
μ (mm^−1^)	0.08
Crystal size (mm)	0.25 × 0.15 × 0.15

Data collection	
Diffractometer	Bruker D8 QUEST PHOTON-III CCD
Absorption correction	Multi-scan (*SADABS*; Krause *et al.*, 2015 ▸)
*T*_min_, *T*_max_	0.973, 0.981
No. of measured, independent and observed [*I* > 2σ(*I*)] reflections	39899, 7913, 4972
*R*_int_	0.081
(sin θ/λ)_max_ (Å^−1^)	0.758

Refinement	
*R*[*F* ^2^ > 2σ(*F* ^2^)], *wR*(*F* ^2^), *S*	0.054, 0.123, 1.01
No. of reflections	7913
No. of parameters	296
H-atom treatment	H atoms treated by a mixture of independent and constrained refinement
Δρ_max_, Δρ_min_ (e Å^−3^)	0.27, −0.17

Computer programs: *APEX3* (Bruker, 2018 ▸), *SAINT* (Bruker, 2013 ▸), *SHELXT2014/5* (Sheldrick, 2015*a* ▸), *SHELXL2018/3* (Sheldrick, 2015*b* ▸), *ORTEP-3 for Windows* (Farrugia, 2012 ▸) and *PLATON* (Spek, 2020 ▸).

## supplementary crystallographic information

### Crystal data

**Table:**

C_28_H_21_N_3_O	*D*_x_ = 1.268 Mg m^−^^3^
*M**_r_* = 415.48	Mo *K*α radiation, λ = 0.71073 Å
Orthorhombic, *P*2_1_2_1_2_1_	Cell parameters from 5592 reflections
*a* = 10.7038 (3) Å	θ = 2.3–27.1°
*b* = 11.6096 (4) Å	µ = 0.08 mm^−^^1^
*c* = 17.5182 (5) Å	*T* = 296 K
*V* = 2176.93 (11) Å^3^	Prism, pale yellow
*Z* = 4	0.25 × 0.15 × 0.15 mm
*F*(000) = 872	

### Data collection

**Table:**

Bruker D8 QUEST PHOTON-III CCD diffractometer	4972 reflections with *I* > 2σ(*I*)
φ and ω scans	*R*_int_ = 0.081
Absorption correction: multi-scan (SADABS; Krause *et al.*, 2015)	θ_max_ = 32.6°, θ_min_ = 2.3°
*T*_min_ = 0.973, *T*_max_ = 0.981	*h* = −16→16
39899 measured reflections	*k* = −17→17
7913 independent reflections	*l* = −26→26

### Refinement

**Table:**

Refinement on *F*^2^	Hydrogen site location: mixed
Least-squares matrix: full	H atoms treated by a mixture of independent and constrained refinement
*R*[*F*^2^ > 2σ(*F*^2^)] = 0.054	*w* = 1/[σ^2^(*F*_o_^2^) + (0.0438*P*)^2^ + 0.242*P*] where *P* = (*F*_o_^2^ + 2*F*_c_^2^)/3
*wR*(*F*^2^) = 0.123	(Δ/σ)_max_ < 0.001
*S* = 1.01	Δρ_max_ = 0.27 e Å^−^^3^
7913 reflections	Δρ_min_ = −0.17 e Å^−^^3^
296 parameters	Extinction correction: SHELXL2018/3 (Sheldrick 2015b), Fc^*^=kFc[1+0.001xFc^2^λ^3^/sin(2θ)]^-1/4^
0 restraints	Extinction coefficient: 0.0098 (17)

### Special details

**Table:**

Geometry. All esds (except the esd in the dihedral angle between two l.s. planes) are estimated using the full covariance matrix. The cell esds are taken into account individually in the estimation of esds in distances, angles and torsion angles; correlations between esds in cell parameters are only used when they are defined by crystal symmetry. An approximate (isotropic) treatment of cell esds is used for estimating esds involving l.s. planes.

### Fractional atomic coordinates and isotropic or equivalent isotropic displacement parameters (Å^2^)

**Table:**

	*x*	*y*	*z*	*U*_iso_*/*U*_eq_	
C1	0.8603 (2)	0.5325 (2)	0.67650 (12)	0.0305 (5)	
C2	0.8260 (2)	0.7274 (2)	0.72668 (12)	0.0301 (5)	
C3	0.7054 (2)	0.7266 (2)	0.69663 (13)	0.0291 (5)	
C4	0.66343 (19)	0.6306 (2)	0.65291 (12)	0.0267 (4)	
C5	0.7394 (2)	0.53749 (19)	0.64189 (12)	0.0276 (4)	
C6	0.7003 (2)	0.43797 (19)	0.59129 (13)	0.0284 (5)	
H6	0.736642	0.367572	0.612719	0.034*	
C7	0.5567 (2)	0.4246 (2)	0.59334 (15)	0.0331 (5)	
H7A	0.531069	0.377784	0.550221	0.040*	
H7B	0.533864	0.383461	0.639483	0.040*	
C8	0.4857 (2)	0.5366 (2)	0.59104 (12)	0.0285 (5)	
C9	0.5374 (2)	0.6316 (2)	0.62044 (12)	0.0288 (5)	
H9	0.492214	0.700033	0.620253	0.035*	
C10	0.6260 (2)	0.8212 (2)	0.71308 (14)	0.0343 (5)	
C11	1.0205 (2)	0.6280 (2)	0.75426 (14)	0.0327 (5)	
C12	1.0273 (2)	0.6001 (3)	0.83030 (15)	0.0468 (7)	
H12	0.955194	0.583755	0.857910	0.056*	
C13	1.1434 (3)	0.5967 (3)	0.86523 (17)	0.0564 (8)	
H13	1.149044	0.579063	0.916902	0.068*	
C14	1.2495 (3)	0.6189 (3)	0.82467 (18)	0.0516 (7)	
H14	1.327119	0.615648	0.848401	0.062*	
C15	1.2409 (2)	0.6460 (3)	0.74895 (18)	0.0580 (8)	
H15	1.313123	0.661550	0.721318	0.070*	
C16	1.1260 (2)	0.6505 (3)	0.71289 (15)	0.0469 (7)	
H16	1.120619	0.668558	0.661272	0.056*	
C17	0.7496 (2)	0.4510 (2)	0.51044 (13)	0.0298 (5)	
C18	0.8123 (3)	0.3613 (2)	0.47579 (16)	0.0469 (7)	
H18	0.825842	0.293484	0.502852	0.056*	
C19	0.8557 (3)	0.3702 (3)	0.40138 (17)	0.0558 (8)	
H19	0.898031	0.308574	0.379378	0.067*	
C20	0.8370 (3)	0.4683 (3)	0.36024 (16)	0.0485 (7)	
H20	0.865482	0.473690	0.310203	0.058*	
C21	0.7759 (3)	0.5586 (3)	0.39356 (16)	0.0518 (7)	
H21	0.762974	0.626088	0.366047	0.062*	
C22	0.7328 (3)	0.5504 (2)	0.46820 (15)	0.0458 (6)	
H22	0.692085	0.612929	0.490148	0.055*	
C23	0.3570 (2)	0.5371 (2)	0.56106 (12)	0.0299 (5)	
C24	0.2781 (2)	0.4429 (2)	0.57364 (14)	0.0357 (5)	
H24	0.307748	0.379405	0.600472	0.043*	
C25	0.1563 (2)	0.4429 (2)	0.54661 (15)	0.0425 (6)	
H25	0.104480	0.380215	0.556168	0.051*	
C26	0.1121 (2)	0.5354 (3)	0.50570 (15)	0.0458 (7)	
H26	0.030803	0.534899	0.486933	0.055*	
C27	0.1884 (3)	0.6286 (3)	0.49262 (16)	0.0473 (7)	
H27	0.158275	0.691099	0.464940	0.057*	
C28	0.3100 (2)	0.6304 (2)	0.52037 (14)	0.0384 (6)	
H28	0.360334	0.694330	0.511654	0.046*	
N1	0.89903 (16)	0.63210 (18)	0.71755 (11)	0.0305 (4)	
N2	0.8714 (2)	0.8181 (2)	0.76538 (14)	0.0438 (6)	
N3	0.5640 (2)	0.8978 (2)	0.72858 (15)	0.0528 (6)	
O1	0.93170 (16)	0.44937 (15)	0.67346 (11)	0.0417 (4)	
H2A	0.825 (3)	0.877 (3)	0.7691 (17)	0.050*	
H2B	0.952 (3)	0.823 (3)	0.7796 (17)	0.050*	

### Atomic displacement parameters (Å^2^)

**Table:**

	*U*^11^	*U*^22^	*U*^33^	*U*^12^	*U*^13^	*U*^23^
C1	0.0284 (10)	0.0349 (12)	0.0283 (11)	0.0014 (10)	−0.0022 (9)	0.0002 (9)
C2	0.0283 (10)	0.0354 (12)	0.0265 (11)	−0.0017 (10)	−0.0019 (9)	−0.0015 (9)
C3	0.0273 (11)	0.0313 (11)	0.0288 (12)	−0.0002 (9)	−0.0019 (9)	−0.0029 (9)
C4	0.0252 (10)	0.0306 (11)	0.0244 (10)	−0.0010 (9)	−0.0010 (8)	0.0021 (9)
C5	0.0256 (10)	0.0297 (11)	0.0275 (10)	−0.0002 (9)	−0.0030 (8)	−0.0002 (9)
C6	0.0283 (10)	0.0258 (11)	0.0309 (11)	0.0002 (9)	−0.0037 (9)	0.0008 (9)
C7	0.0314 (11)	0.0312 (12)	0.0367 (13)	−0.0035 (10)	0.0012 (10)	−0.0007 (10)
C8	0.0266 (10)	0.0336 (12)	0.0252 (10)	−0.0015 (9)	−0.0006 (8)	0.0016 (9)
C9	0.0257 (10)	0.0308 (12)	0.0297 (11)	0.0029 (9)	−0.0022 (8)	−0.0011 (9)
C10	0.0316 (11)	0.0380 (13)	0.0333 (12)	0.0007 (11)	−0.0041 (10)	−0.0063 (10)
C11	0.0272 (11)	0.0396 (12)	0.0314 (11)	−0.0006 (10)	−0.0050 (9)	−0.0020 (10)
C12	0.0380 (13)	0.069 (2)	0.0339 (14)	−0.0065 (13)	−0.0054 (11)	0.0098 (13)
C13	0.0567 (18)	0.072 (2)	0.0406 (16)	−0.0060 (16)	−0.0210 (14)	0.0100 (14)
C14	0.0356 (13)	0.0614 (18)	0.0578 (18)	0.0037 (14)	−0.0196 (13)	−0.0071 (15)
C15	0.0275 (12)	0.092 (2)	0.0543 (18)	−0.0056 (15)	−0.0007 (12)	−0.0048 (17)
C16	0.0349 (13)	0.073 (2)	0.0332 (13)	−0.0020 (14)	−0.0001 (11)	0.0001 (13)
C17	0.0274 (10)	0.0313 (11)	0.0308 (11)	−0.0036 (10)	−0.0017 (8)	−0.0038 (9)
C18	0.0605 (17)	0.0344 (14)	0.0460 (15)	0.0030 (13)	0.0124 (13)	−0.0011 (12)
C19	0.071 (2)	0.0434 (16)	0.0534 (17)	−0.0014 (15)	0.0249 (16)	−0.0146 (14)
C20	0.0507 (16)	0.0587 (18)	0.0360 (13)	−0.0198 (14)	0.0086 (12)	−0.0090 (13)
C21	0.0619 (19)	0.0519 (17)	0.0414 (15)	0.0014 (15)	0.0081 (13)	0.0109 (13)
C22	0.0553 (16)	0.0407 (14)	0.0413 (15)	0.0086 (13)	0.0102 (12)	0.0041 (12)
C23	0.0285 (11)	0.0344 (12)	0.0268 (10)	−0.0030 (10)	−0.0008 (9)	−0.0031 (9)
C24	0.0324 (12)	0.0371 (13)	0.0376 (13)	−0.0042 (10)	−0.0010 (10)	0.0012 (11)
C25	0.0321 (13)	0.0485 (15)	0.0467 (15)	−0.0094 (12)	0.0001 (10)	−0.0037 (13)
C26	0.0311 (12)	0.0607 (18)	0.0456 (15)	−0.0020 (13)	−0.0088 (11)	−0.0070 (14)
C27	0.0456 (15)	0.0501 (16)	0.0461 (15)	0.0035 (14)	−0.0166 (12)	0.0061 (13)
C28	0.0372 (13)	0.0389 (13)	0.0391 (13)	−0.0050 (11)	−0.0069 (10)	0.0033 (11)
N1	0.0257 (9)	0.0371 (11)	0.0287 (10)	−0.0004 (8)	−0.0049 (7)	−0.0020 (8)
N2	0.0345 (11)	0.0425 (12)	0.0545 (14)	0.0002 (10)	−0.0121 (11)	−0.0171 (11)
N3	0.0507 (13)	0.0523 (15)	0.0553 (16)	0.0171 (12)	−0.0070 (12)	−0.0123 (12)
O1	0.0358 (9)	0.0394 (10)	0.0499 (11)	0.0092 (8)	−0.0108 (8)	−0.0036 (9)

### Geometric parameters (Å, º)

**Table:**

C1—O1	1.232 (3)	C14—H14	0.9300
C1—N1	1.424 (3)	C15—C16	1.384 (4)
C1—C5	1.430 (3)	C15—H15	0.9300
C2—N2	1.343 (3)	C16—H16	0.9300
C2—N1	1.364 (3)	C17—C18	1.380 (3)
C2—C3	1.395 (3)	C17—C22	1.383 (4)
C3—C10	1.418 (3)	C18—C19	1.388 (4)
C3—C4	1.424 (3)	C18—H18	0.9300
C4—C5	1.367 (3)	C19—C20	1.363 (4)
C4—C9	1.464 (3)	C19—H19	0.9300
C5—C6	1.515 (3)	C20—C21	1.367 (4)
C6—C17	1.519 (3)	C20—H20	0.9300
C6—C7	1.545 (3)	C21—C22	1.390 (4)
C6—H6	0.9800	C21—H21	0.9300
C7—C8	1.507 (3)	C22—H22	0.9300
C7—H7A	0.9700	C23—C28	1.390 (3)
C7—H7B	0.9700	C23—C24	1.399 (3)
C8—C9	1.338 (3)	C24—C25	1.387 (3)
C8—C23	1.475 (3)	C24—H24	0.9300
C9—H9	0.9300	C25—C26	1.375 (4)
C10—N3	1.142 (3)	C25—H25	0.9300
C11—C16	1.367 (3)	C26—C27	1.375 (4)
C11—C12	1.373 (4)	C26—H26	0.9300
C11—N1	1.451 (3)	C27—C28	1.390 (4)
C12—C13	1.386 (4)	C27—H27	0.9300
C12—H12	0.9300	C28—H28	0.9300
C13—C14	1.364 (4)	N2—H2A	0.84 (3)
C13—H13	0.9300	N2—H2B	0.90 (3)
C14—C15	1.366 (4)		
			
O1—C1—N1	118.5 (2)	C14—C15—H15	119.6
O1—C1—C5	125.1 (2)	C16—C15—H15	119.6
N1—C1—C5	116.39 (19)	C11—C16—C15	119.0 (2)
N2—C2—N1	119.2 (2)	C11—C16—H16	120.5
N2—C2—C3	122.1 (2)	C15—C16—H16	120.5
N1—C2—C3	118.7 (2)	C18—C17—C22	117.2 (2)
C2—C3—C10	118.2 (2)	C18—C17—C6	120.3 (2)
C2—C3—C4	120.0 (2)	C22—C17—C6	122.5 (2)
C10—C3—C4	121.8 (2)	C17—C18—C19	121.3 (3)
C5—C4—C3	120.48 (19)	C17—C18—H18	119.3
C5—C4—C9	120.0 (2)	C19—C18—H18	119.3
C3—C4—C9	119.5 (2)	C20—C19—C18	120.7 (3)
C4—C5—C1	120.7 (2)	C20—C19—H19	119.7
C4—C5—C6	121.44 (19)	C18—C19—H19	119.7
C1—C5—C6	117.83 (19)	C19—C20—C21	119.1 (3)
C5—C6—C17	111.96 (19)	C19—C20—H20	120.5
C5—C6—C7	109.75 (19)	C21—C20—H20	120.5
C17—C6—C7	112.15 (19)	C20—C21—C22	120.5 (3)
C5—C6—H6	107.6	C20—C21—H21	119.7
C17—C6—H6	107.6	C22—C21—H21	119.7
C7—C6—H6	107.6	C17—C22—C21	121.2 (3)
C8—C7—C6	114.47 (19)	C17—C22—H22	119.4
C8—C7—H7A	108.6	C21—C22—H22	119.4
C6—C7—H7A	108.6	C28—C23—C24	118.1 (2)
C8—C7—H7B	108.6	C28—C23—C8	121.6 (2)
C6—C7—H7B	108.6	C24—C23—C8	120.3 (2)
H7A—C7—H7B	107.6	C25—C24—C23	120.9 (2)
C9—C8—C23	121.4 (2)	C25—C24—H24	119.6
C9—C8—C7	119.52 (19)	C23—C24—H24	119.6
C23—C8—C7	119.0 (2)	C26—C25—C24	120.1 (3)
C8—C9—C4	121.6 (2)	C26—C25—H25	120.0
C8—C9—H9	119.2	C24—C25—H25	120.0
C4—C9—H9	119.2	C27—C26—C25	119.8 (2)
N3—C10—C3	177.8 (3)	C27—C26—H26	120.1
C16—C11—C12	121.0 (2)	C25—C26—H26	120.1
C16—C11—N1	119.9 (2)	C26—C27—C28	120.6 (3)
C12—C11—N1	119.1 (2)	C26—C27—H27	119.7
C11—C12—C13	118.9 (3)	C28—C27—H27	119.7
C11—C12—H12	120.6	C23—C28—C27	120.4 (2)
C13—C12—H12	120.6	C23—C28—H28	119.8
C14—C13—C12	120.7 (3)	C27—C28—H28	119.8
C14—C13—H13	119.6	C2—N1—C1	123.46 (18)
C12—C13—H13	119.6	C2—N1—C11	119.22 (19)
C13—C14—C15	119.5 (3)	C1—N1—C11	117.25 (19)
C13—C14—H14	120.2	C2—N2—H2A	117 (2)
C15—C14—H14	120.2	C2—N2—H2B	122 (2)
C14—C15—C16	120.8 (3)	H2A—N2—H2B	119 (3)
			
N2—C2—C3—C10	−5.1 (4)	C5—C6—C17—C18	129.5 (2)
N1—C2—C3—C10	173.7 (2)	C7—C6—C17—C18	−106.6 (3)
N2—C2—C3—C4	177.0 (2)	C5—C6—C17—C22	−51.4 (3)
N1—C2—C3—C4	−4.2 (3)	C7—C6—C17—C22	72.5 (3)
C2—C3—C4—C5	1.9 (3)	C22—C17—C18—C19	−0.6 (4)
C10—C3—C4—C5	−176.0 (2)	C6—C17—C18—C19	178.5 (3)
C2—C3—C4—C9	−178.9 (2)	C17—C18—C19—C20	−0.2 (5)
C10—C3—C4—C9	3.3 (3)	C18—C19—C20—C21	0.7 (5)
C3—C4—C5—C1	2.5 (3)	C19—C20—C21—C22	−0.3 (5)
C9—C4—C5—C1	−176.7 (2)	C18—C17—C22—C21	1.0 (4)
C3—C4—C5—C6	−175.9 (2)	C6—C17—C22—C21	−178.1 (3)
C9—C4—C5—C6	4.9 (3)	C20—C21—C22—C17	−0.6 (5)
O1—C1—C5—C4	175.4 (2)	C9—C8—C23—C28	38.7 (3)
N1—C1—C5—C4	−4.4 (3)	C7—C8—C23—C28	−145.5 (2)
O1—C1—C5—C6	−6.1 (3)	C9—C8—C23—C24	−140.8 (2)
N1—C1—C5—C6	174.06 (19)	C7—C8—C23—C24	35.0 (3)
C4—C5—C6—C17	94.7 (2)	C28—C23—C24—C25	−0.4 (4)
C1—C5—C6—C17	−83.7 (2)	C8—C23—C24—C25	179.2 (2)
C4—C5—C6—C7	−30.5 (3)	C23—C24—C25—C26	1.1 (4)
C1—C5—C6—C7	151.0 (2)	C24—C25—C26—C27	−0.9 (4)
C5—C6—C7—C8	41.4 (3)	C25—C26—C27—C28	0.0 (4)
C17—C6—C7—C8	−83.8 (3)	C24—C23—C28—C27	−0.6 (4)
C6—C7—C8—C9	−29.4 (3)	C8—C23—C28—C27	179.9 (2)
C6—C7—C8—C23	154.7 (2)	C26—C27—C28—C23	0.8 (4)
C23—C8—C9—C4	177.7 (2)	N2—C2—N1—C1	−179.0 (2)
C7—C8—C9—C4	1.9 (3)	C3—C2—N1—C1	2.2 (3)
C5—C4—C9—C8	11.5 (3)	N2—C2—N1—C11	4.2 (3)
C3—C4—C9—C8	−167.8 (2)	C3—C2—N1—C11	−174.6 (2)
C16—C11—C12—C13	1.0 (4)	O1—C1—N1—C2	−177.8 (2)
N1—C11—C12—C13	−179.7 (3)	C5—C1—N1—C2	2.0 (3)
C11—C12—C13—C14	−1.0 (5)	O1—C1—N1—C11	−0.9 (3)
C12—C13—C14—C15	0.7 (5)	C5—C1—N1—C11	178.9 (2)
C13—C14—C15—C16	−0.4 (5)	C16—C11—N1—C2	−100.4 (3)
C12—C11—C16—C15	−0.6 (5)	C12—C11—N1—C2	80.2 (3)
N1—C11—C16—C15	−179.9 (3)	C16—C11—N1—C1	82.5 (3)
C14—C15—C16—C11	0.3 (5)	C12—C11—N1—C1	−96.8 (3)

### Hydrogen-bond geometry (Å, º)

*Cg*1, *Cg*4 and *Cg*5 are the centroids of the 1,2-dihydropyridine ring (N1/C1–C5) and the C17–C22 and C23–C28 phenyl rings, respectively.

**Table:**

*D*—H···*A*	*D*—H	H···*A*	*D*···*A*	*D*—H···*A*
N2—H2*B*···O1^i^	0.90 (3)	2.09 (3)	2.813 (3)	136 (3)
C7—H7*B*···N3^ii^	0.97	2.54	3.391 (4)	146
C13—H13···*Cg*5^iii^	0.93	2.74	3.576 (3)	149
C26—H26···*Cg*4^iv^	0.93	2.83	3.729 (3)	162
C16—H16···*Cg*5^v^	0.93	2.97	3.603 (3)	126
C20—H20···*Cg*1^vi^	0.93	2.96	3.514 (3)	120

Symmetry codes: (i) −*x*+2, *y*+1/2, −*z*+3/2; (ii) −*x*+1, *y*−1/2, −*z*+3/2; (iii) −*x*+3/2, −*y*+1, *z*+1/2; (iv) *x*−1, *y*, *z*; (v) *x*+1, *y*, *z*; (vi) −*x*+3/2, −*y*+1, *z*−1/2.
